# The Select Committee on the Medical Examination of Recruits

**Published:** 1917-07-14

**Authors:** 


### THE SELECT COMMITTEE ON THE MEDICAL EXAMINATION OF RECRUITS.

### Some Notes on the Evidence.

In the earlier stages of the war, and prior to the days of conscription, the medical examination of recruits came in for a good deal of criticism, and the criticism invariably challenged the rejection of men who were alleged to be well fitted for service in the Army. Now that compulsion is exercising its sway, criticism runs entirely in the opposite direction, and the accusation against the doctors is that they accept men who are unfit for military duties and discipline. It is out of this latter contention that the Select Committee of the House of Commons has come into existence. Later there will arrive the duty of reviewing the Committee's report. In the meanwhile it is advisable to take note of some of the facts and opinions which have been given in evidence. From the medical point of view, and more particularly from the point of view of the medical recruiting officer, no statement carries more importance than the view expressed by Colonel Galloway, that under the existing arrangement the profession has been asked to accomplish an impossible task. The reference is to the demand for the sorting of men into classes or groups not less than nine in number, and having boundary lines impossible of exact and precise definition. In the very nature of things this must mean differences of opinion among the examiners, and, apart from the inevitable conflict and the prejudicial influence of this on the public, time must be wasted at the Boards in unnecessary discussion. There is, however, evidence which shows that not all of the medical differences can be explained in this fashion. Thus Sir John Bland-Sutton, as a member of one of the Special or Appeal Medical Boards, submitted figures which showed that of 3,449 men whose cases, on appeal, came under his review, 1,622 were placed in a lower category and an additional 336 were rejected as quite unfit for service. Moreover, of the 336 thus rejected 135 had previously been certified as fit for active service, that is they had been placed in Class A. Whether a particular man should be in B 2 or C 2 may not unreasonably lead to some difference of opinion, but between "fit for general service" and "total rejection" is a gap which no charity can bridge. In each of these instances someone has blundered and has blundered badly, and for such mistakes the recruiting officers, on one Board or the other, must carry the discredit. That the Medical Boards generally do their work well, and in circumstances by no means always easy, is not in dispute, and is challenged only by those who are familiar solely with the exceptions to this rule. What the Select Committee has got to find out is whether any means can be devised to lessen the number of such exceptions.

On two points some conflict of view is to be found between witnesses who represent respectively the military and the civilian outlook. The first of these differences refers to the range of time exercised by the classification attached to the man when he enters the Army. The military view evidently is that such classification has merely temporary value and that it may be changed, and even changed with but little delay, when the Army has once secured its man. On the other hand is the statement of Sir John Bland-Sutton that in his view the classification fixed by his Board was to hold good during the man's term of service. In practice it is certain that the former doctrine has prevailed, and men have been pushed up, and sometimes rapidly, into the more strenuous groups; and this is one of the grounds of fairly widespread and bitter complaint. The practice is obviously inconsistent with a principle allowed in evidence by the military witnesses—namely, that the majority of members of a recruiting board ought to be civilian practitioners. If this is true for the classification of a man on entering the Army it must be true when it is proposed to change his classification, whereas under the present practice the re-examination must obviously be made by the military doctors. There are certainly instances where men feel that under this scheme they have been dealt with unfairly, and it is easy to see how such a sense of injustice arises. Military emergencies excuse, even if they do not justify, many rough proceedings, but to breed a sense of bad faith is a harmful thing for any organisation.

A second point of difference came to light in reference to the contention that any man who was fit to work in civil life was fit to do the same work in the Army. The soldiers apparently are content with this generalisation, but Sir John Bland-Sutton, though his illustration of the stockbroker with the weak heart was not a very apt one, will have none of it. And surely common sense is with Sir John, for it would be news to hear that a degree of domestic care and comfort and cooking which will carry on a dyspeptic clerk for many a year, can be provided in camps and tented fields. Quite naturally the soldiers wish to secure their man and feel annoyed if he escapes them. Equally the man who is satisfied that he is unfit for Army service desires to avoid the claim. Between the two the doctors have to decide, and the task is often a difficult one.

An amusing point in the evidence of the medical experts is the contempt they throw on certificates granted by other experts who do not happen to be adorned with a temporary uniform. The family doctor's opinion is always welcome, it is said (he will be surprised to hear it), but a single examination even by an expert is of little value. If this argument is true, the whole proceeding of the medical examination of recruits is obviously under condemnation, for in every instance the verdict is framed after a single examination. Thus the witnesses belittle in others what they themselves are now doing in the Army, and what in less heroic days they preached as consulting physicians and surgeons and advisers to insurance companies. Self-satisfaction is evidently one of the effects of an official atmosphere.

